# High genetic risk for depression as an independent risk factor for mortality in patients referred for coronary angiography

**DOI:** 10.3389/fcvm.2023.1125151

**Published:** 2023-06-26

**Authors:** Robert M. Krämer, Angela P. Moissl, Stefan Lorkowski, Bernhard K. Krämer, Terho Lehtimäki, Binisha H. Mishra, Pashupati P. Mishra, Jan Leipe, Winfried März, Marcus E. Kleber, Bertram Müller-Myhsok, Graciela E. Delgado

**Affiliations:** ^1^ Department for Children and Adolescent Psychiatry, Central Institute for Mental Health Mannheim, Medical Faculty Mannheim of the University of Heidelberg, Mannheim, Germany; ^2^ Vth Department of Medicine, University Medicine Mannheim, Medical Faculty Mannheim of the University of Heidelberg, Mannheim, Germany; ^3^ Institute of Nutritional Sciences, Friedrich Schiller University, Jena, Germany; ^4^ Competence Cluster for Nutrition and Cardiovascular Health (nutriCARD), Halle-Jena-Leipzig, Germany; ^5^ Center for Preventive Medicine and Digital Health Baden-Württemberg (CPDBW), Medical Faculty Mannheim, Heidelberg University, Mannheim, Germany; ^6^ European Center for Angioscience ECAS, Faculty of Medicine of the University of Heidelberg, Mannheim, Germany; ^7^ Department of Clinical Chemistry, Fimlab Laboratories and Finnish Cardiovascular Research Center Tampere, Faculty of Medicine and Health Technology, Tampere University, Tampere, Finland; ^8^ Synlab Academy, SYNLAB Holding Deutschland GmbH, Mannheim and Augsburg, Germany; ^9^ SYNLAB MVZ Humangenetik Mannheim, Mannheim, Germany; ^10^ Department of Translational Research in Psychiatry, Max Planck Institute for Psychiatry, Munich, Germany; ^11^ Department of Health Data Science, University of Liverpool, Liverpool, United Kingdom

**Keywords:** depression, coronary artery disease, all-cause mortality, genetic risk score, cardiovascular mortality

## Abstract

**Background:**

Different observations have suggested that patients with depression have a higher risk for a number of comorbidities and mortality. The underlying causes have not been fully understood yet.

**Aims:**

The aim of our study was to investigate the association of a genetic depression risk score (GDRS) with mortality [all-cause and cardiovascular (CV)] and markers of depression (including intake of antidepressants and a history of depression) in the Ludwigshafen Risk and Cardiovascular Health (LURIC) study involving 3,316 patients who had been referred for coronary angiography.

**Methods and results:**

The GDRS was calculated in 3,061 LURIC participants according to a previously published method and was found to be associated with all-cause (*p* = 0.016) and CV mortality (*p* = 0.0023). In Cox regression models adjusted for age, sex, body mass index, LDL-cholesterol, HDL-cholesterol, triglycerides, hypertension, smoking, and diabetes mellitus, the GDRS remained significantly associated with all-cause [1.18 (1.04–1.34, *p* = 0.013)] and CV [1.31 (1.11–1.55, *p* = 0.001)] mortality. The GDRS was not associated with the intake of antidepressants or a history of depression. However, this cohort of CV patients had not specifically been assessed for depression, leading to marked underreporting. We were unable to identify any specific biomarkers correlated with the GDRS in LURIC participants.

**Conclusion:**

A genetic predisposition for depression estimated by a GDRS was independently associated with all-cause and CV mortality in our cohort of patients who had been referred for coronary angiography. No biomarker correlating with the GDRS could be identified.

## Introduction

Approximately 18% of the world population is affected by depression making it a common psychiatric disorder (1, 2). According to the World Health Organization (WHO), since 2004, depression has been ranked as the third major contributor to the overall global burden of disease and as the most common cause of disease burden among females (3). In 2017, the WHO ranked depression as the leading cause of disability worldwide with a prevalence of >427 million people of all ages (4).

Individuals with depression have been reported to experience increased risks of various somatic diseases (5–13). Longitudinal studies have reported increased risks of asthma (11–13), diabetes mellitus (8, 10), cardiovascular (CV) disease (5, 7), Parkinson's disease (6), and dementia (9) for patients with depression. Using data from the National Registry of Denmark, an increased risk for up to 30 different medical conditions as well as for mortality following a mood disorder diagnosis (including depression) has been reported (14–16). In a pooled analysis of individual–participant data from 563,255 participants in 22 prospective cohorts from the Emerging Risk Factors Collaboration (ERFC) and the UK Biobank, depressive symptoms were associated with a risk of incident CV disease after multivariate adjustment for CV risk factors (17). In addition, depressive symptoms were associated with all-cause and CV mortality, and an even stronger association was observed for non-cancer, non-CV mortality (17).

Genetic predisposition to depression has been extensively studied, resulting in 102 independent variants significantly associated (*p* < 5 × 10^−8^) with a recent genome-wide meta-analysis of 807,553 individuals from the UK Biobank, 23andMe, and the Psychiatric Genomics Consortium (PGC) (18). Addition of data from the Million Veteran Program resulted in identification of additional 77 genetic loci associated with depression (19). According to Howard et al., the weighted genetic depression risk score (GDRS) (18) has previously been shown to be associated with suicidal ideation and planning among Mexican adolescents (*n* = 1,128) and young adults (*n* = 437) (20).

Howard et al. estimated the single nucleotide polymorphism (SNP)-based heritability of depression in their study to be 8.9% using a broad definition of depression. For a narrow case definition of major depressive disorder, considerably higher estimated heritability of 29%–34% has been reported (21).

Mendelian randomization analyses have consistently shown that a genetic liability to depression was associated with a higher risk for coronary artery disease (CAD), heart failure, and small vessel stroke and that this association is partly mediated by cardiovascular risk factors such as diabetes mellitus, blood lipids, or smoking (22, 23). However, genetic factors underlying depression have rarely been studied for their impact on cardiovascular and all-cause mortality. The aim of our study was to analyze the associations between the weighted genetic depression risk score (18) and morbidity and mortality in CV high-risk patients referred for coronary angiography enrolled in the Ludwigshafen Risk and Cardiovascular Health (LURIC) study. Furthermore, we evaluated a possible association between the GDRS and the intake of antidepressants or a history of depression in our cohort of CV patients, who had not specifically been assessed for depression.

## Materials and methods

### Subjects

The LURIC study is a monocentric hospital-based cohort study that recruited 3,316 patients of German ancestry at the Ludwigshafen Heart Center in South-West Germany. All participants were referred for elective coronary angiography due to established or suspected CAD. Participants with acute illnesses other than acute coronary syndromes, such as malignancy or other chronic non-cardiac diseases, within the previous 5 years were excluded.

The ethics committee of the “Landesärztekammer Rheinland-Pfalz” [LURIC, #837.255.97 (1394)] approved the study which was conducted in accordance with the Declaration of Helsinki. Informed written consent was obtained from all participants.

### Laboratory methods

Fasting blood samples were obtained by venipuncture at study entry (24). A summary of analytic methods has been previously reported (24).

Triglycerides and cholesterol were quantified with enzymatic reagents from WAKO (Neuss, Germany) on a WAKO 30R analyzer. Lipoproteins were separated using a combined ultracentrifugation–precipitation method (β-quantification) as follows: plasma was ultracentrifuged using a Beckman LM-8 ultracentrifuge in 100-μl volumes with a VT-51.2 rotor (Beckman Coulter) at a density of *d* = 1.0063 kg/L (30,000 rpm for 18 h). very-low-density lipoprotein (VLDL) in the supernatant was removed. In the remainder, low-density lipoprotein (LDL) and intermediate-density lipoprotein (IDL) were precipitated with phosphotungstic acid/MgCl_2_. Centrifugation (10,000 rpm for 5 min) was then performed to separate precipitated LDL (and IDL) from HDL in the remainder. Apolipoproteins were measured by turbidimetry (Rolf Greiner BioChemica). Lipoprotein(a) concentrations were measured in plasma using the LPA Test (Rolf Greiner Biochimica, Flacht, Germany). The creatinine level was measured using the CREA assay (Roche, Germany) on a Hitachi 717 analyzer. The glucose level was measured with an enzymatic assay on a Hitachi 717 analyzer. The measurement of glycated hemoglobin (Hb) was done using an immunoassy (hemoglobin A1c UNIMATE 5; Hoffmann-La Roche, Grenzach-Whylen, Germany). The levels of high-sensitive C-reactive protein (CRP) and cystatin C were measured by immunonephelometry (N-High-Sensitive CRP; N-Latex Cystatin C, Dade Behring, Marburg, Germany) using a Behring nephelometer II. The N-terminal pro-B-type natriuretic peptide (NT-proBNP) level was measured by electro-chemoluminescence on an Elecsys 2010 (Roche Diagnostics).

### Definition of clinical variables and endpoints

According to the American Heart Association (AHA) classification, CAD was defined as the presence of visible lumen narrowing (>20% stenosis) in at least 1 of 15 coronary segments. According to the guidelines of the American Diabetes Association from 2010, diabetes mellitus was defined as an increased level of fasting glucose (≥126 mg/dl) and/or increased level of glucose after a 2-h glucose tolerance test (≥200 mg/dl) and/or increased level of glycated HbA1c (≥6.5%) and/or a history of diabetes mellitus (25). According to the 2018 European Society of Cardiology/European Society of Hypertension Guidelines for the management of arterial hypertension, hypertension was defined as systolic blood pressure of ≥140 and/or diastolic blood pressure of ≥90 mm Hg or as a history of hypertension (26). The 2012 Chronic Kidney Disease Edidemiology Collaboration creatinin-cystatin C equation was used to estimate the glomerular filtration rate (27).

Patients were categorized as having a diagnosis of presumptive depression if they met one of two criteria: (1) self-reported depression (*n* = 3) or (2) intake of antidepressant drugs (*n* = 85). In total, 88 study participants were classified as probably suffering from depression. Information on the vital status of study participants was requested from local registries. The median follow-up time was 9.9 (8.5–10.7) years. Death certificates, medical records of local hospitals, and autopsy data were independently reviewed by two experienced clinicians who were blinded to patient characteristics and who classified the causes of death. CV mortality included sudden cardiac death (*n* = 236, 7.7%), fatal myocardial infarction (*n* = 92, 3.0%), death due to congestive heart failure (*n* = 129, 4.2%), death after intervention to treat CAD (*n* = 25, 0.8%), fatal stroke (*n* = 59, 1.9%), and other causes of death due to CAD (*n* = 16, 0.5%).

### Genotyping, imputation, and calculation of genetic risk score of depression

Genotyping was performed by using the Affymetrix Human SNP Array 6.0. Samples with a low call rate of <95% and samples for which the calculated sex from the array analysis did not match the sex from the database were excluded. A total of 3,061 samples were available for further analyses. Variants with a call rate of less than 0.98, Hardy–Weinberg equilibrium of *p* < 5 × 10^−4^, and minor allele frequency (MAF) of <0.01 were removed leaving 686,195 SNPs for imputation. Genotype imputation was performed using the 1,000 Genomes Project phase I version 3 reference panel and IMPUTE2.

We extracted 101 of the 102 SNPs delineated in Howard et al. (18) as genome-wide significant from our genetic dataset and calculated the weighted genetic risk score using PLINK v2 and the weightings by Howard et al. (18). In brief, the weighted GDRS was defined as the sum of the number of depression-risk-increasing alleles at each locus multiplied by the respective β-coefficient reported from the meta-analysis.

### Statistical analyses

Continuous data are shown as the mean and standard deviation (SD) when normally distributed or as the median and 25th and 75th percentile for non-normally distributed variables. Categorical data are presented as percentages. Statistical differences between groups and continuous variables were determined using analysis of variance. Non-normally distributed variables (triglycerides, hsCRP, NT-pro-BNP, and alcohol) were log-transformed before entering analysis. The chi-square test was used for categorical variables.

Time-to-event analyses were performed using multivariable Cox proportional hazard models. Multivariable adjustment was carried out as indicated in order to investigate, whether a genetic predisposition for depression serves as an independent predictor for mortality. The study participants were split into quartiles of GDRS or into two groups according to the median of the GDRS. A competing risk model according to the method proposed by Fine and Gray (28) was calculated for cause-specific mortality using the finegray and coxph functions as implemented in the R package “survival” version 3.4-0.

Survival curves for the different groups were calculated by Kaplan–Meier analyses using the R package “survminer” version 0.4.3. We also adjusted the distribution of possible confounders by inverse probability weighting, thereby balancing the subgroups for the confounding variables [age, sex, diabetes mellitus, smoking, low-density lipoprotein cholesterol (LDL-C), high-density lipoprotein cholesterol (HDL-C), hypertension, body mass index (BMI), and triglycerides]. Weights were calculated using logistic regression models. A weighted Cox model was calculated, and we report the result of the robust score test as implemented in the coxph function in R that corresponds to a log-rank test corrected for weighting. The proportional hazard assumption was checked by examination of scaled Schoenfeld residuals.

Hazard ratio plots were created using the R package “rms” version 6.4-1. Restricted cubic splines with three knots (at the tenth, fiftieth, and ninetieth percentile of the distribution) were calculated using the rcs function from the rms R package.

All tests were two-sided, and a *p*-value of <0.05 was considered statistically significant. All analyses were carried out using SPSS version 27 and R version 4.2.2 (http://www.r-project.org).

## Results

**Figure 1:**
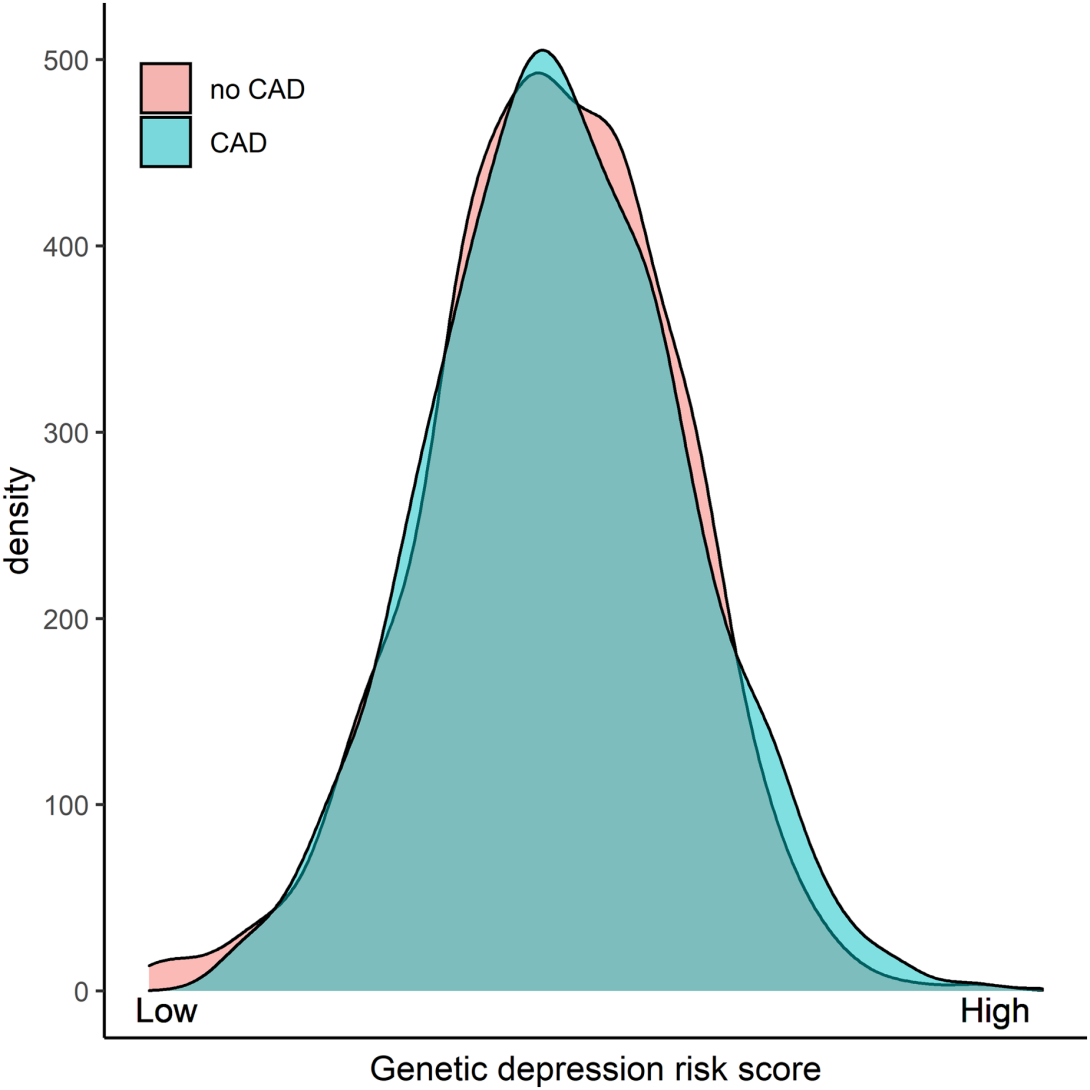
Distribution of the GDRS in patients with or without CAD. GDRS, genetic depression risk score; CAD, coronary artery disease.

The GDRS was approximately normally distributed in LURIC participants with only slight differences between participants with and without CAD at study entry (Figure 1).

**Figure 2:**
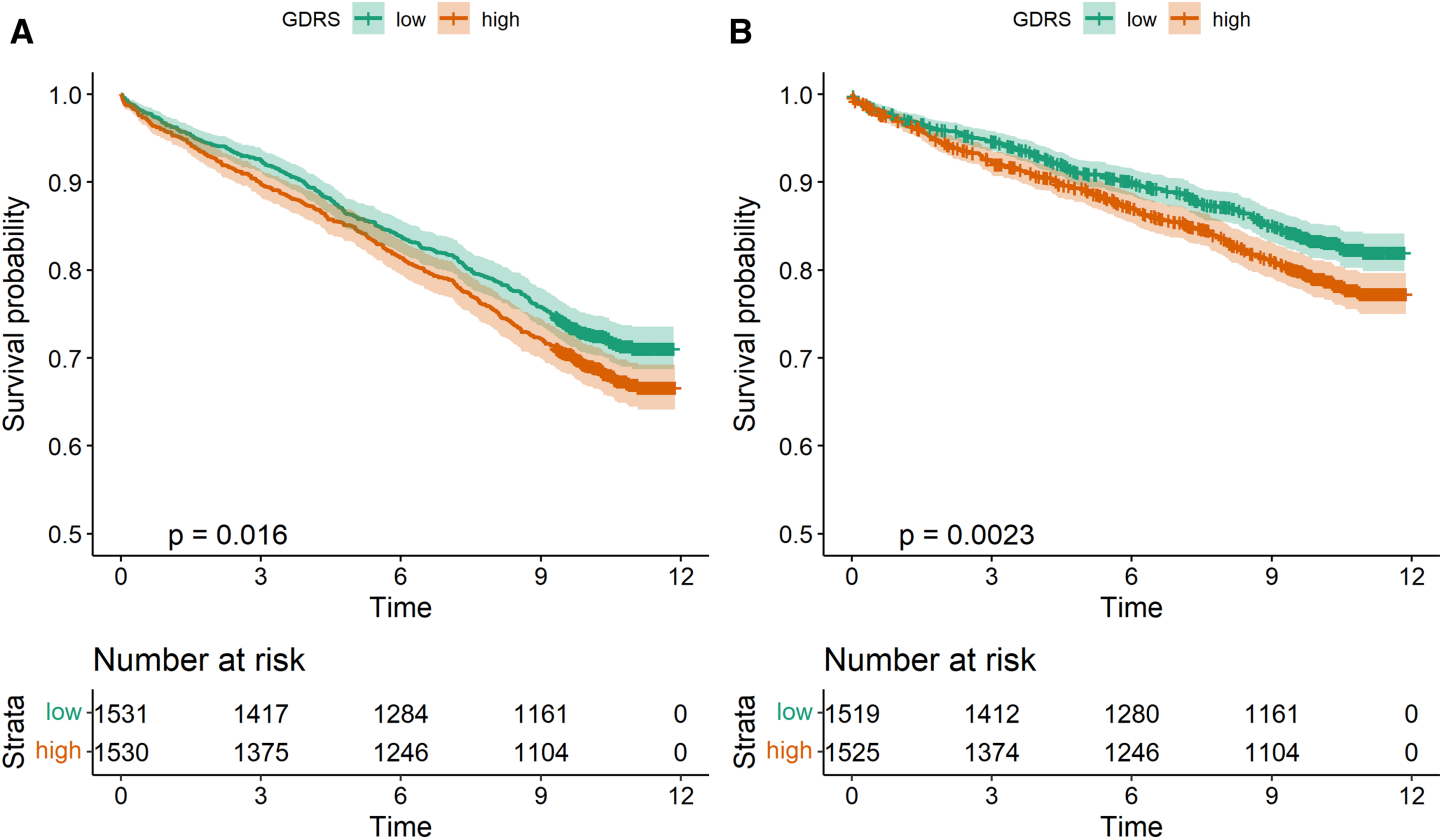
Kaplan–Meier curves for all-cause mortality (**A**) and CV mortality (**B**) according to GDRS groups. CV, cardiovascular; GDRS, genetic depression risk score.

**Figure 3:**
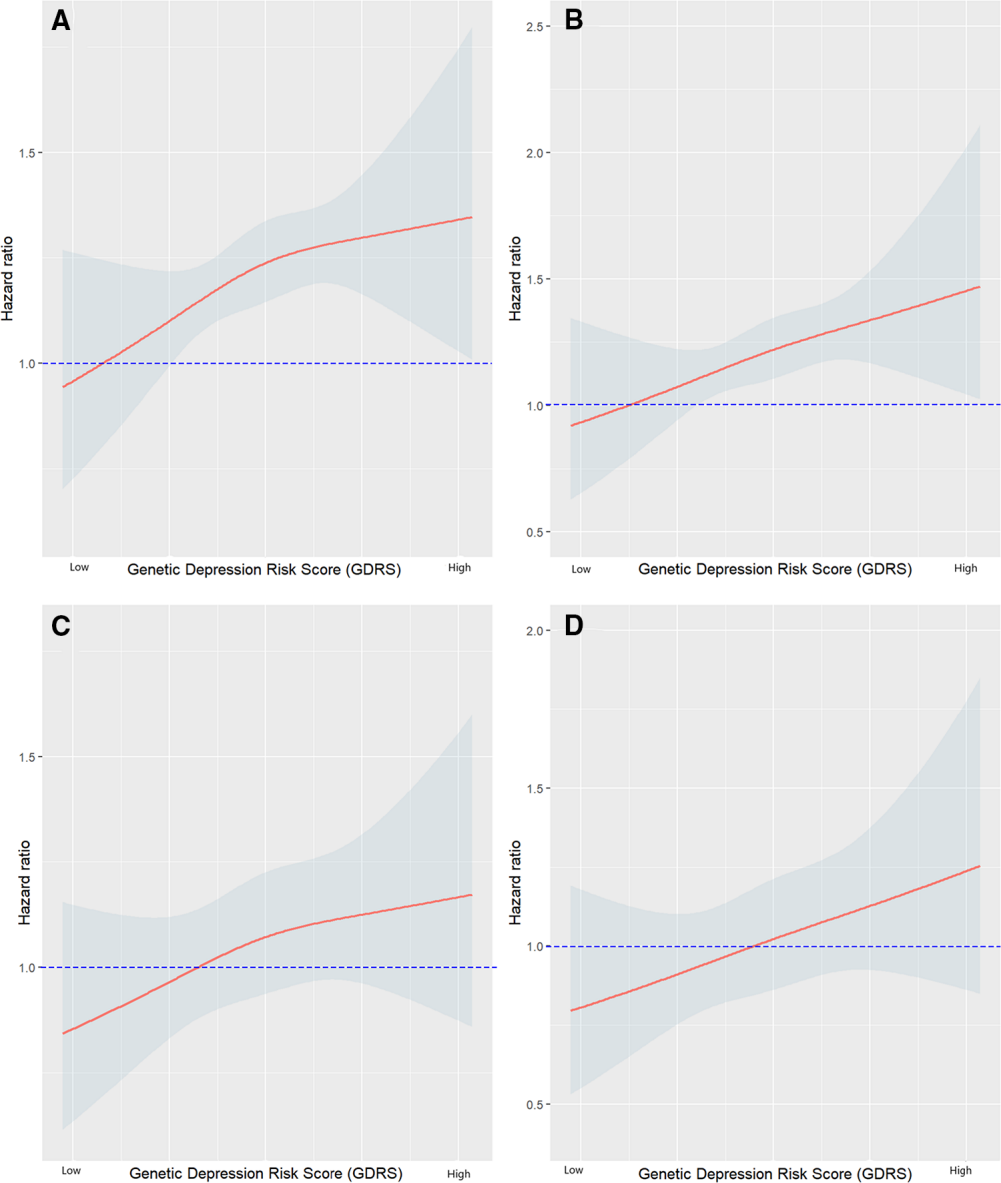
Hazard ratio plots showing the association of GDRS with mortality (**A**) all-cause mortality adjusted for age and sex; (**B**) CV mortality adjusted for age and sex; (**C**) all-cause mortality adjusted for age, sex, BMI, LDL-C, HDL-C, log triglycerides (TG), hypertension, smoking, and diabetes mellitus; (**D**) CV mortality adjusted for age, sex, BMI, LDL-C, HDL-C, log TG, hypertension, smoking, and diabetes mellitus. GDRS, genetic depression risk score; CV, cardiovascular; BMI, body mass index.

Next, we investigated whether the GDRS was associated with long-term outcomes in our cohort. Therefore, we split the cohort into quartiles of GDRS and analyzed the association with all-cause and CV mortality. The Kaplan–Meier curves, especially for all-cause mortality, show that the lower two quartiles and the upper two quartiles cluster together (Supplementary Figures S1A,B), and therefore we decided to split our cohort into two groups according to the median of the GDRS for further analyses (Figures 2A,B). A higher GDRS was significantly associated with increased all-cause (*p* = 0.016) and CV (*p* = 0.0023) mortality. The same trend was observed when the GDRS was modeled as a restricted cubic spline (Figure 3).

When we subdivided the LURIC cohort according to gender, GDRS was significantly associated with all-cause (*p* = 0.011) and CV (*p* = 0.0011) mortality only in men, but not in women (*p* = 0.97 and *p* = 0.76) (Supplementary Figure S3). No significant association between GDRS and diagnosed or suspected depression was found (*p* = 0.385) (Table 1).

**Table 1:** Study characteristics according to GDRS (mean and SD or median and 25th to 75th percentile).

Variable	All (*n* = 3,061)	Low GDRS (*n* = 1,531)	High GDRS (*n* = 1,530)	p*
Age (years)	62.7 (10.6)	62.8 (10.7)	62.6 (10.6)	0.535
Female sex (%)	30	30.2	29.7	0.822
BMI (kg/m^2^)	27.5 (4.03)	27.5 (4.11)	27.5 (3.94)	0.978
LDL-C (mg/dl)	117 (34.5)	117 (34.5)	116 (34.4)	0.245
HDL-C (mg/dl)	38.7 (10.8)	38.6 (10.4)	38.8 (11.2)	0.628
TG (mg/dl)	147 (109–201)	146 (110–200)	147 (108–201)	0.987
HbA1c (%)	6.31 (1.24)	6.29 (1.23)	6.32 (1.26)	0.541
SysBP (mmHg)	141 (23.6)	141 (23.9)	141 (23.3)	0.788
DiaBP (mmHg)	80.9 (11.4)	80.9 (11.3)	80.9 (11.6)	0.947
eGFR (ml/min/1.73 m^2^)	81.7 (20.2)	81.4 (20.1)	82.0 (20.5)	0.484
hsCRP (mg/L)	3.46 (1.32–8.74)	3.6 (1.34–9.01)	3.36 (1.3–8.48)	0.628
NT-pro-BNP (pg/ml)	297 (108–882)	305 (109–881)	292 (107–894)	0.791
Alcohol (g eth/d)	3.08 (0–24)	3.22 (0–24)	3 (0–24)	0.346
Coronary artery disease (%)	79.0	78.6	79.4	0.632
Hypertension (%)	72.7	71.6	73.8	0.184
T2DM (%)	40.4	40.5	40.3	0.924
Smokers (%)	23.2	21.9	24.4	0.101
Self-reported depression or intake of antidepressants (%)	2.9	3.1	2.6	0.385
Intake of sedatives (%)	4.3	4.4	4.2	0.793
All-cause mortality (%)	29.8	27.8	31.8	0.016
Cardiovascular mortality (%)	18.4	16.3	20.5	0.002
Non-cardiovascular mortality (%)	11.0	10.9	11.0	0.984

GDRS, genetic depression risk score; BMI, body mass index; BP, blood pressure; eGFR, estimated glomerular filtration rate; GDRS, genetic depression risk score; hsCRP, high-sensitivity CRP; T2DM, type 2 diabetes mellitus.

*Low GDRS vs. high GDRS: *t*-test for continuous variables (non-normally distributed variables were log-transformed before entering the analysis) and chi-square test for categorical variables.

When we subdivided our LURIC cohort into two groups according to the median of the GDRS, the severity of CAD was not significantly different between groups, though there was a trend for more severe CAD with a higher GDRS (*p* = 0.07; chi-square test) (Supplementary Table S1).

Study characteristics according to GDRS groups or quartiles are shown in Table 1 and Supplementary Table S2. There was no statistical difference for any of the examined biomarkers between participants with high or low GDRS or between the quartiles.

In order to investigate whether a genetic predisposition for depression serves as an independent predictor for mortality, we calculated Cox regression models and adjusted the analyses for CV risk factors. Table 2 shows the results of the different models. For CV mortality, Fine and Gray's competing risk model was used in treating non-cardiovascular death as competing events. Additionally adding probable depression to model 2 only marginally changed the results (data not shown).

**Table 2:** Cox regression analyses for all-cause and cause-specific mortality.

GDRS	All-cause mortality		Cardiovascular death		Other causes of death	
	HR (95%CI)	*p*	HR (95%CI)	*p*	HR (95%CI)	*p*
Crude						
Low	1		1		1	
High	1.17 (1.03–1.34)	0.016	1.29 (1.09–1.52)	0.003	1.01 (0.812–1.25)	0.955
Model 1						
Low	1		1		1	
High	1.22 (1.07–1.39)	0.003	1.34 (1.14–1.59)	0.001	1.01 (0.819–1.25)	0.899
Model 2						
Low	1		1		1	
High	1.21 (1.06–1.38)	0.004	1.35 (1.15–1.60)	0.001	0.990 (0.799–1.23)	0.924

HR, hazard ratio; CI, confidence interval.

According to Fine and Gray, for the calculation of the hazard for cardiovascular mortality and other causes of death, a competing risk model has been calculated; model 1, adjusted for age and sex; model 2, additionally adjusted for BMI, LDL-C, HDL-C, log triglycerides, hypertension, smoking, and diabetes mellitus; GDRS, genetic depression risk score.

**Figure 4:**
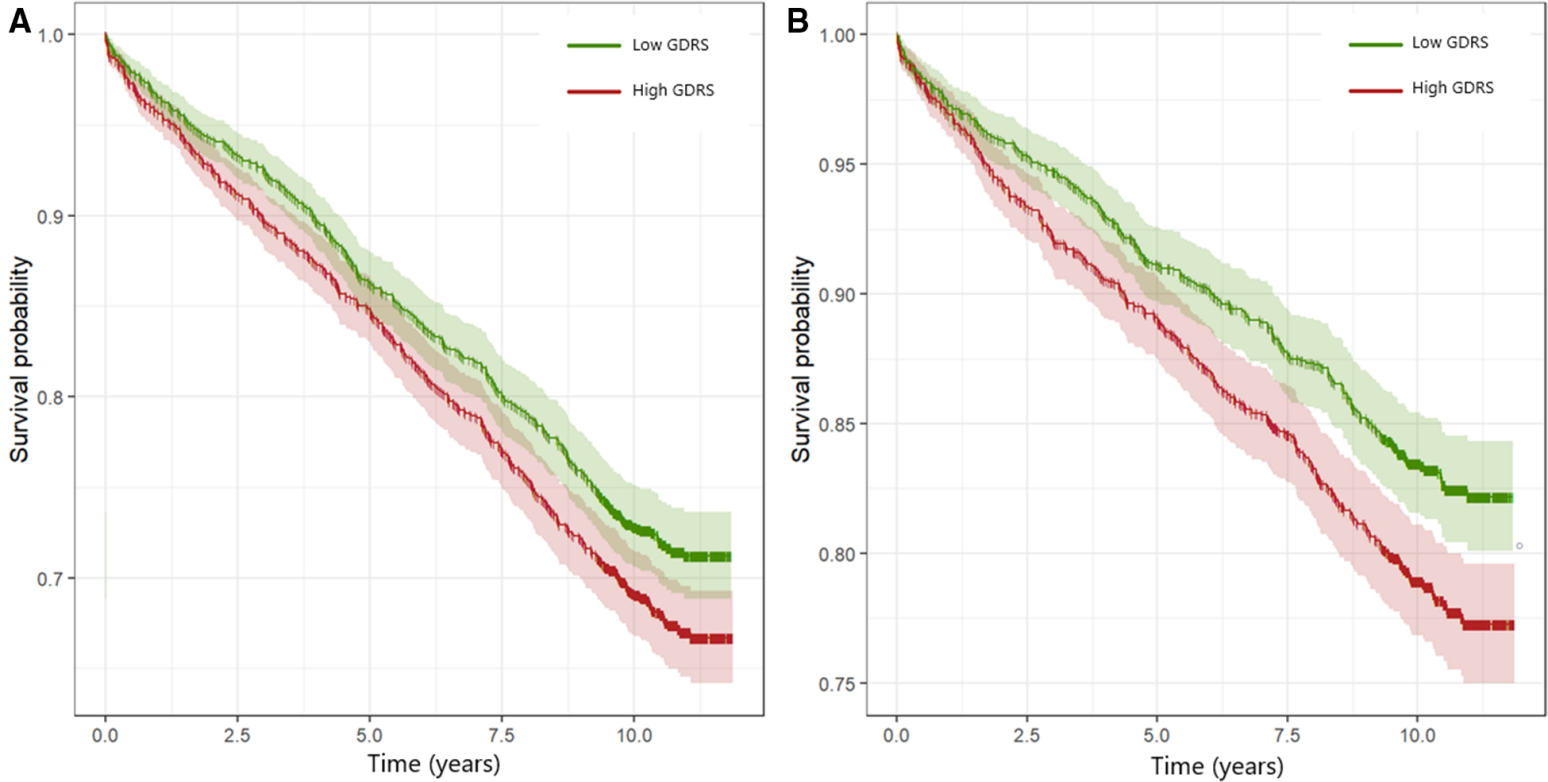
Adjusted survival curves for all-cause mortality (**A**) and CV mortality (**B**). The groups with high and low GDRS were balanced for age, sex, body mass index, LDL-C, HDL-C, log TG, smoking, hypertension, and diabetes mellitus by inverse variance weighting. Hazard ratios (95% confidence interval, *p*-value) for the groups with high GDRS compared with the groups with low GDRS were 1.18 (1.04–1.34, *p* = 0.013) and 1.31 (1.11–1.55, *p* = 0.001) for all-cause and CV mortality, respectively. GDRS, genetic depression risk score; CV, cardiovascular.

Figure 4 shows adjusted survival curves in which the two groups have been balanced for potential confounding variables. The analyses suggested an increased risk of CV mortality as assessed by hazard ratios (HR) in patients with higher GDRS scores but no increased risk for non-cardiovascular death.

## Discussion

The most important finding of our study was that a higher GDRS was predictive of CV and all-cause mortality both in crude analyses and in models adjusted for CV risk factors. Specifically, the GDRS was significantly associated with an approximately 30% higher risk of CV mortality (HR range from 1.30 to 1.34) and an approximately 20% higher risk of all-cause mortality (HR range from 1.17 to 1.21). The magnitude of these effects implies clinical relevance and suggests that CV mortality is affected to a larger extent than other causes of death. Using a competing risk model, there was no significant association of the GDRS with non-cardiovascular death. This finding is somewhat at variance with the data from Harshfield et al. (17), who reported similar HRs for depression symptoms and the risk of CV (1.09 ERFC/1.18 UK Biobank) and all-cause mortality (1.09 ERFC/1.15 UK Biobank) but higher HRs for non-cancer, non-CV mortality (1.17 ERFC/1.31 UK Biobank). These differences between our study and the analysis from Harshfield et al. (17) can likely be explained by different phenotypes (depression symptoms vs. GDRS) and baseline differences in study cohorts: while LURIC studied patients at intermediate to high cardiovascular risk, ERFC and UK Biobank are population-based cohorts.

Other population-based cohorts have also found similar effects of depression on CV and all-cause mortality, including a recent study of two large population-based cohorts from China in which depression was associated with CV mortality (HR 1.22 and 1.32) and all-cause mortality (HR 1.32 or 1.17) in multivariate analyses (29). Interestingly, this association was much stronger in men than in women (only significant for all-cause mortality in one cohort with a HR of 1.19) although more than half of study participants were females. These latter findings correspond well with the results of our analysis, in which a significant effect of the GDRS on CV and all-cause mortality was also restricted to men; however, in LURIC, only 30% of participants were females.

With regard to underlying mechanisms causing the association between genetic predisposition for depression and all-cause and CV mortality in our study, a recent large meta-analysis that investigated a bi-directional causal association between depression and cardiovascular diseases using a Mendelian randomization study was of special interest (22). Genetic predisposition to depression was found to be causally associated with CAD and myocardial infarction, but not with atrial fibrillation, while the genetic risk for CV disease was not associated with depression (22). The genetic risk of depression may mediate its negative effects on CV disease via more smoking and higher lipid levels (22). These findings were confirmed in another meta-analysis using overlapping data sources (23) implicating type 2 diabetes and smoking as important mediators for CAD/myocardial infarction (MI). Similarly, in another large meta-analysis, Torgersen et al. (30) found a polygenic overlap between loci associated with depression and loci associated with CAD, BMI, systolic blood pressure, lipids, type 2 diabetes, and C-reactive protein. However, in our study in patients at intermediate to high CV risk, there were only slight trends for more smokers, and more hypertensives, but lower LDL-cholesterol in the higher GDRS quartiles (Supplementary Table S1). Furthermore, a significant association between the GDRS and CV and all-cause mortality persisted in our study after adjustment for CV risk factors including smoking and lipids. These latter findings may be due to the fact that in LURIC baseline risk factors such as LDL-cholesterol and blood pressure had been modified by lipid lowering and/or antihypertensive treatment in the majority of patients (24). In the Young Finns cohort study, the GDRS was insignificantly associated (*p* = 0.136) with a carotid media thickness of >90th percentile in an age-, sex-, and BMI-adjusted analysis (unpublished observation). This finding argues against a very marked effect of GDRS on atherosclerosis, which is in line with our findings in LURIC that the GDRS was not significantly associated with atherosclerosis.

Since LURIC was not planned to specifically study depression, reporting of clinical diagnoses was presumably largely incomplete with self-reported depression in only 3 (0.1%) patients and use of antidepressants or sedatives in another 88 (2.9%) or 132 (4.3%) patients. Only the use of antidepressants and the self-reported diagnosis of depression can be taken as some evidence to imply the presence of depression, whereas the use of sedatives, which are most often used for sleeping disorders in Germany, is rather nonspecific for making a diagnosis of depression. Nevertheless, also antidepressants may have been used for other reasons such as pain control or may have been prescribed by the general practitioner with a nonspecific indication. This underreporting/underassessment of depression in LURIC may very well explain why the clinical diagnosis of depression did not correlate with the GDRS. Furthermore, between July 1997 and January 2000 when patients were included into LURIC, there still were considerable societal barriers in Germany to being at ease to openly report depression, as indicated by only 0.1% of our patients reporting depression. In contrast, an analysis of 14,746 unselected primary care patients presenting in 412 practices in Germany on 4 April 2000 found a point prevalence of depression of 10.9% [11.9% in females, 9.4% in males; most cases (71%) were moderate to severe], when using the Depression Screening Questionnaire (DSQ) (31). Only 55% of these patients with depression had been correctly diagnosed by their primary care physician, and, of these, fewer than 60% had received an antidepressant. Given these numbers on contemporary depression prevalence and antidepressant treatment rates in Germany, the true prevalence of self-reported depression in LURIC is estimated to be 100-fold higher than assessed. Therefore, these data and seeming group differences are grossly unreliable and should not be considered as valid in relation to the GDRS.

### Strengths and limitations

All LURIC participants were of European origin (limiting genetic heterogeneity) and were recruited at a tertiary referral center. Therefore, our findings may not be representative for a random population sample or applicable to other ethnicities. Furthermore, at study enrolment, participants were not specifically asked whether they ever suffered from depression and therefore the reporting of clinical diagnoses was presumably largely incomplete. However, the major strengths of the LURIC cohort are the precise clinical and metabolic characterization of the participants including the availability of coronary angiograms and the availability of a complete 10-year follow-up on mortality.

### Conclusion

Depression as assessed by the GDRS is independently associated with all-cause and CV mortality in a CV high-risk cohort. No biomarker correlating with the GDRS could be identified in our analysis.

## Data Availability

The data analyzed in this study is subject to the following licenses/restrictions: Due to the articles of Ludwigshafen Risk and Cardiovascular Health (LURIC) Study GmbH, which needs to acknowledge the German Data Protection Act and the consent given by the study participants, data cannot be released to the public domain. Interested researchers are invited to address their request or proposal to access the dataset to Kai Grunwald (kai.grunwald@weitnauer.net) or to the principal investigator of the LURIC study WM (winfried.maerz@luric-online.de).
