# Supplementary material for: High genetic risk for depression as an independent risk factor for mortality in patients referred for coronary angiography
Source: Front Cardiovasc Med. 2023 Jun 26;10:1125151. doi: 10.3389/fcvm.2023.1125151 (PMC10330785; doi:10.3389/fcvm.2023.1125151)
Supplement: Supplementary file 1 [file Datasheet1.docx]

**Supplement**

**Supplemental Table 1: Severity of CAD given as 0 (0-49% stenosis), 1 (1-vessel disease), 2 (2-vessel disease), 3 (3-vessel disease) in the two patient groups with higher and lower GDRS (median split)**

| CAD: Number of affected vessels |  | | | |
| --- | --- | --- | --- | --- |
|  | Low GDRS | | High GDRS | |
|  | N | % | N | % |
| 0 | 483 | 52.3% | 440 | 47.7% |
| 1 | 295 | 50.3% | 291 | 49.7% |
| 2 | 297 | 50.7% | 289 | 49.3% |
| 3 | 442 | 47.9% | 481 | 52.1% |
| Chi-square-test, P value = 0.070 (linear by linear) | | | | |

**Supplemental Table 2: Study characteristics according to GDRS quartiles (mean and SD or median and 25^th^ to 75^th^ percentile)**

|  | **GDRS quartile** | | | |  |
| --- | --- | --- | --- | --- | --- |
| **Variable** | **1st** | **2nd** | **3rd** | **4th** | **P*** |
| Age (years) | 63(10.6) | 62.6(10.8) | 62.7(10.5) | 62.5(10.7) | 0.781 |
| Female sex (%) | 29.1 | 31.2 | 30.2 | 29.3 | 0.789 |
| BMI (kg/m2) | 27.3(3.85) | 27.6(4.35) | 27.4(3.97) | 27.5(3.91) | 0.365 |
| LDL-C (mg/dl) | 118(35.6) | 116(33.4) | 117(36) | 114(32.8) | 0.150 |
| HDL-C (mg/dl) | 38.7(10.5) | 38.4(10.3) | 39.1(11.5) | 38.4(10.9) | 0.455 |
| TG (mg/dl) | 145(108-195) | 150(111-204) | 145(108-201) | 148(109-202) | 0.525 |
| HbA1c (%) | 6.29(1.24) | 6.29(1.22) | 6.36(1.31) | 6.28(1.2) | 0.524 |
| Systolic blood pressure (mmHg) | 141(24.6) | 141(23.1) | 141(23.1) | 142(23.5) | 0.866 |
| Diastolic blood pressure (mmHg) | 80.8(11.7) | 81(10.9) | 80.9(11.5) | 81(11.7) | 0.987 |
| eGFR (ml/min/1.73m2) | 81(20.7) | 81.9(19.4) | 81.2(20.4) | 82.7(20.5) | 0.365 |
| hsCRP (mg/L) | 3.4(1.25-9.1) | 3.79(1.43-8.78) | 3.42(1.36-8.26) | 3.32(1.25-8.53) | 0.867 |
| NT-proBNP (pg/mL) | 318(109-888) | 296(109-864) | 291(113-911) | 293(103-876) | 0.735 |
| Alcohol (g eth/d) | 3.08(0-24) | 3.36(0-24) | 3(0-24) | 3(0-24) | 0.758 |
| Coronary artery disease (%) | 78.6 | 78.7 | 78.6 | 80.3 | 0.815 |
| Hypertension (%) | 71 | 72.2 | 73.5 | 74.1 | 0.531 |
| T2DM (%) | 38.8 | 42.2 | 41.8 | 38.7 | 0.324 |
| Smokers (%) | 22.5 | 21.3 | 23 | 25.9 | 0.180 |
|  |  |  |  |  |  |

*t-test for continuous variables (non-normally distributed variables were log transformed before entering the analysis) and chi-square test for categorical variables


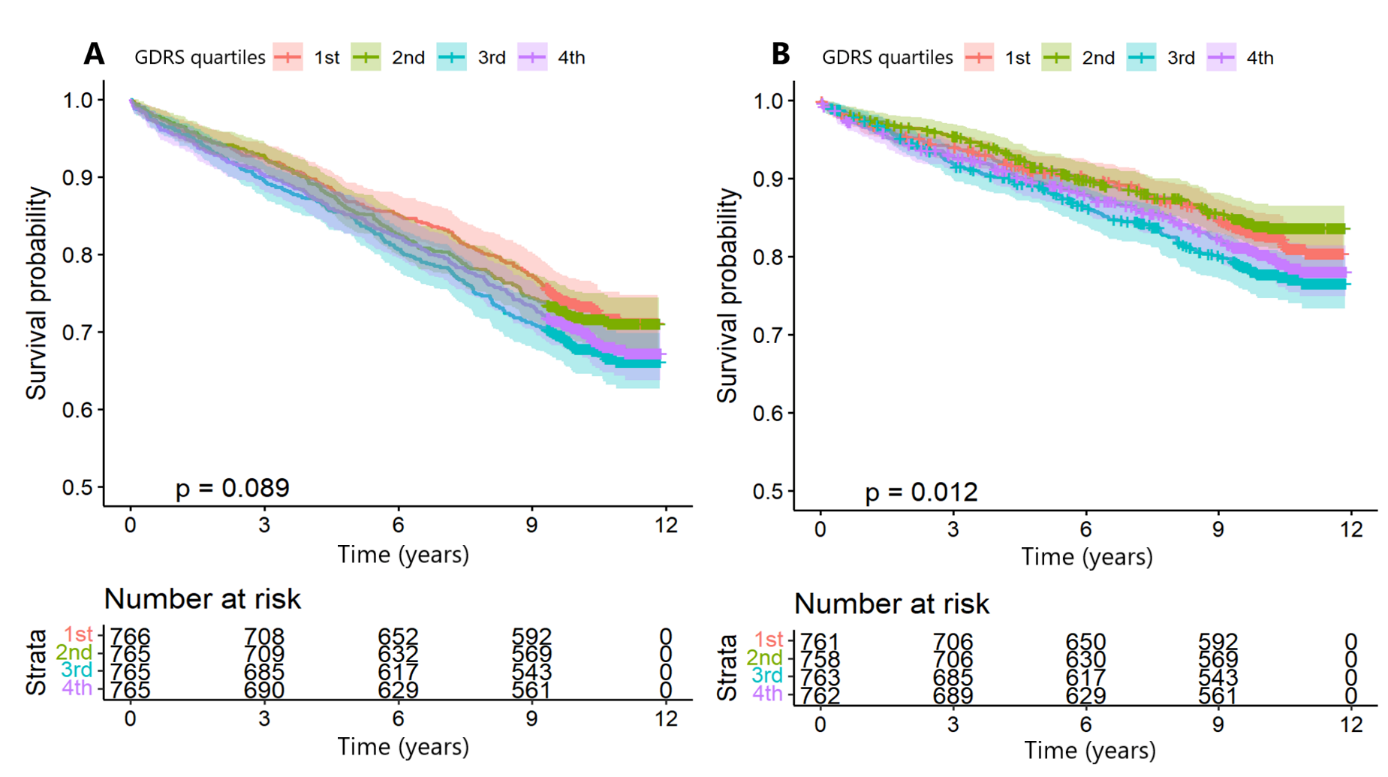


**Supplemental Figure 1:** Kaplan-Meier curves for all-cause mortality (A) and CV mortality (B) according to GDRS quartiles.


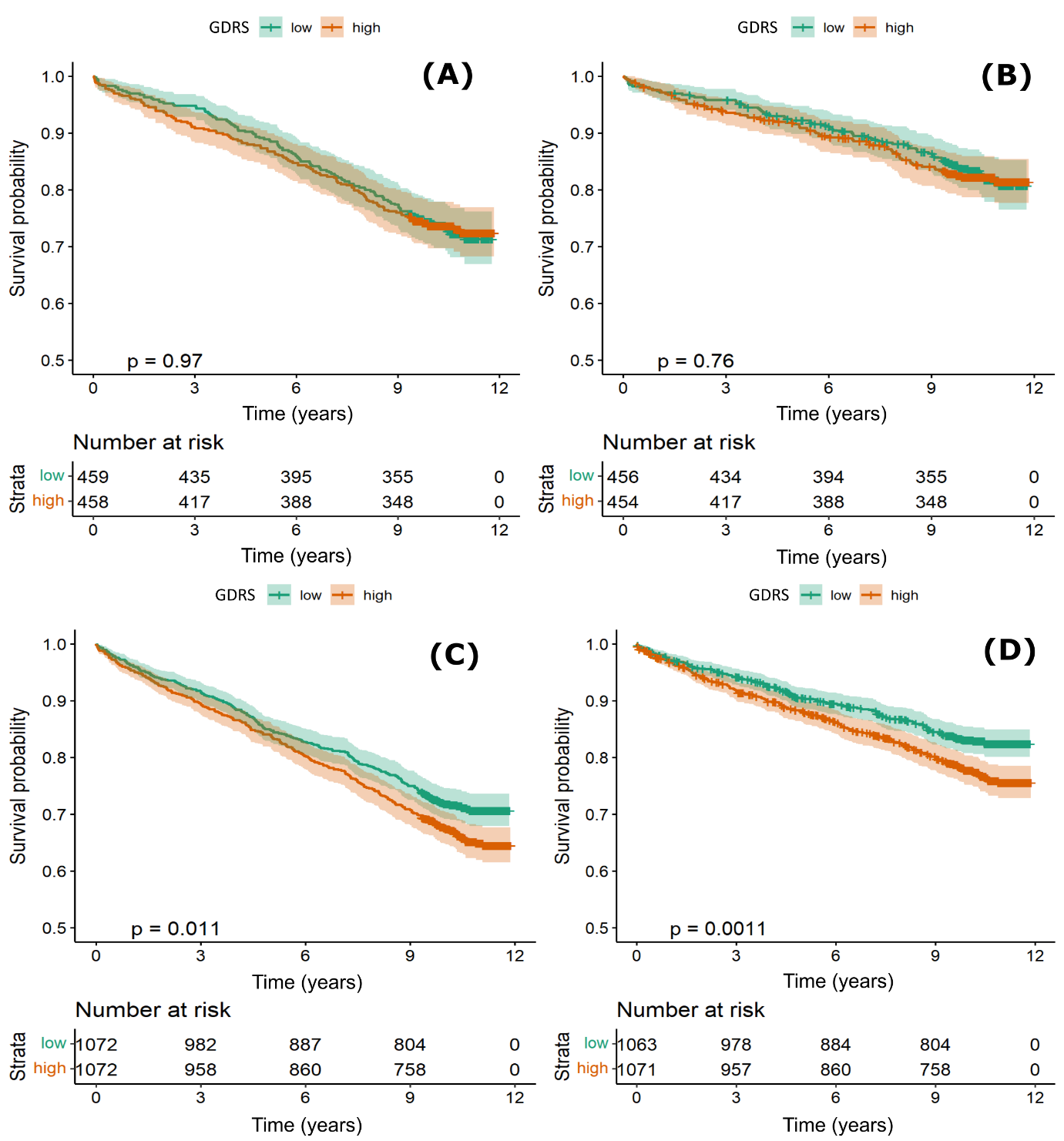


**Supplemental Figure 2:** Gender-specific Kaplan-Meier curves according to GDRS median split: **A)** all-cause mortality (women) **B)** cardiovascular mortality (women) **C**) all-cause mortality (men) **D)** cardiovascular mortality (men).
